# Atypical Presentation of Longstanding Overt Ventriculomegaly in Adults: A Case Report

**DOI:** 10.7759/cureus.58533

**Published:** 2024-04-18

**Authors:** Ernesto Navarro Garcia, Hiba Al-Rubaye, Brooke Norton, Javeria Sahib Din

**Affiliations:** 1 Nanotechnology, University of Central Florida, Orlando, USA; 2 Neuroscience, St George’s University, St. George’s, GRD; 3 Neurology, The Brooklyn Hospital Center, Brooklyn, USA; 4 Neuroscience, St. George’s University School of Medicine, St. George’s, GRD; 5 Psychiatry, Kings County Hospital Center, Brooklyn, USA

**Keywords:** atypical manifestation, normal-pressure hydrocephalus, longstanding overt ventriculomegaly in adults (lova), adult hydrocephalus, ventriculomegaly

## Abstract

Hydrocephalus involves the enlargement of the ventricular system due to increased cerebrospinal fluid. This condition often presents with ventriculomegaly, associated with cognitive decline, gait disturbances, visual changes, and other neurological symptoms. In adults, hydrocephalus may result from longstanding overt ventriculomegaly in adults (LOVA), characterized by macrocephaly, subnormal IQ, urinary incontinence, and gait issues. In a recent case report, a 52-year-old Hispanic female displayed similar predisposing factors and radiological findings for a LOVA diagnosis. Despite the absence of focal neurological deficits or typical complaints, she experienced a breakthrough seizure episode after years without incident.

## Introduction

Hydrocephalus is a neurological disorder marked by the abnormal accumulation of cerebrospinal fluid (CSF) in the brain, causing ventricular enlargement [1]. It can manifest in two main forms: communicating hydrocephalus, where CSF flow is unobstructed, and non-communicating hydrocephalus, caused by an obstruction in CSF circulation [2]. In adults, chronic hydrocephalus falls under chronic hydrocephalus in adults (CHiA), covering all non-acute forms in adulthood [3]. Idiopathic normal-pressure hydrocephalus (iNPH) is a significant subtype, featuring enlarged cerebral ventricles, urinary incontinence, cognitive impairment, and gait apraxia [4].

Hydrocephalus may also lead to increased intracranial pressure, with symptoms such as visual changes and headaches. Distinguishing between idiopathic (iNPH) and secondary (sNPH) forms is vital, with iNPH mostly affecting older adults and sNPH possibly resulting from various causes [5]. However, definitive diagnostic findings for NPH remain elusive, often leading to over-suspecting and under-confirming based on positive shunting responses.

In iNPH, ventricular enlargement involves changes in CSF dynamics and motile cilia function. Genetic mutations, aging, alcohol consumption, sleep apnea, hypertension, and electrolyte imbalances have been implicated in its development [6,7]. Abnormal CSF dynamics increase oscillatory shear stress, affecting ciliary function and causing ventricular enlargement. Understanding these mechanisms is crucial for targeted treatment.

Ventriculomegaly’s heterogeneity ranges from asymptomatic cases to life-threatening conditions. Four typical presentations include incidental findings, highly symptomatic cases, mild symptoms such as headache and nausea, and late symptomatic cases resembling NPH [8]. Seizures may also indicate underlying pathology [9,10].

Other causes of hydrocephalus and ventriculomegaly in adults could be attributed to longstanding overt ventriculomegaly (LOVA) which develops in childhood and arrests before becoming clinically detectable in the fifth or sixth decade of life, as well as a complication of hemodialysis [11-13]. Per reported data, LOVA is characterized by macrocephaly, subnormal IQ, urinary incontinence, and gait disturbances which become more prevalent in the later decades of life [14]. The signs and symptoms of LOVA closely resemble those of NPH and idiopathic intracranial hypertension. Here, we present an atypical case of a patient whose history and radiological findings were consistent with those found in LOVA but did not present with neurological deficits.

## Case presentation

A 52-year-old Hispanic female had a medical history of well-controlled seizures since childhood, scoliosis, asthma, pre-diabetes, history of alcohol, and drug use of crack cocaine. Previous surgical history was notable for a ventriculoperitoneal shunt 40 years ago. Per family historian, she had hydrocephalus as a child, which required the ventriculoperitoneal shunt. Since then, there had been no complications until the current seizure episode. The patient was brought in via emergency medical services for a witnessed episode of generalized tonic-clonic seizure followed by five minutes of loss of consciousness. Per witnesses, there was no tongue biting or incontinence but there was a reported fall causing her to hit her head on the left frontal side. Further history from the patient noted that she was unable to remember the episode itself, the last thing she was able to recall was after the episode when the emergency services arrived (suggestive of loss of consciousness). She also endorsed good insight regarding her episodes by describing that she was aware of when an episode was coming because she could not speak and stared into space, as described by her witnessing peers. The patient also endorsed some memory deficits within the last year, describing them as having trouble remembering where she had placed objects and forgetting the names of familiar faces. Otherwise, she denied sensory or motor disturbances before the event. The patient notes that her last seizure was “some” years ago, and she endorsed compliance with medications, levetiracetam 500 mg BID, and topiramate 200 mg OD, but had now followed up with neurology in two years.

Generally, the patient was pleasant, cooperative, and was a good historian. She was able to describe her previous medical history in detail, describe her medications including dosing and frequencies, as well as describe the episode that brought her into the emergency department. Neurologically, the patient was alert and oriented to person, place, and time. Cranial examination of nerves 2-12 was unremarkable, except for mild inability of the left eye to medially rotate during the convergence test. Pupils were equal, round, and reactive to light and accommodation. Bilateral upper and lower extremity strength was 5/5, and bilateral upper and lower extremities had intact sensation. Reflexes were normal throughout, with a negative Babinski sign. The patient was able to ambulate properly without any assistance. Gait was biomechanically appropriate. During this time, the patient denied any prior episodes of urinary incontinence, visual changes, weakness, paresthesia, or memory changes. The patient stated that she could perform activities of daily living independently and was working on rehabilitation from polysubstance (as stated above) use disorder by being eight months sober from alcohol and eight months clean from crack/cocaine.

At the emergency department, the patient was given 1,000 mg of levetiracetam, intravenous fluids, and acetaminophen for pain control and was taken for computed tomography (CT) of the head without contrast. During this time, the patient reported a minor headache attributed to the fall and generalized muscle ache. No further seizure episodes occurred at this time. Physical examination of the patient was unremarkable except for notable ecchymosis on the left forehead and tenderness to palpation of the left shoulder and arm.

Laboratory findings were unremarkable except for elevated creatine kinase of 173 U/L and chloride of 114 mmol/L. Levetiracetam and topiramate serum levels were within the reference range of 29.0 µg/mL and 10.7 µg/mL, respectively, as shown in Table 1.

**Table 1:** Laboratory workup at the time of admission. The table shows all pertinent labs that could have been involved in the pathogenesis of the acute seizure episode, including electrolytes, glucose, and medication levels. Further lab testing during the hospitalization remained within normal limits until discharge. Stand-out lab values include creatine kinase of 173 U/L, which was normal after seizure due to muscle breakdown, and chloride of 114 mmol/L without anion gap acidosis.

Labs	Value	Range
Creatine kinase	173 U/L	30–135 U/L
Levetiracetam	29.0 µg/mL	Random 12–46 µg/mL
Topiramate	10.7 µg/mL	Random 4–20 µg/mL
Sodium	140 mmol/L	136–145 mmol/L
Potassium	3.8 mmol/L	3.5–5.0 mmol/L
Calcium	8.5 µg/dL	8.4–10.2 µg/dL
Phosphorus	4.4 µg/dL	2.3–4.7 µg/dL
Magnesium	2.3 µg/dL	1.6–2.6 µg/dL
Chloride	114 mmol/L	98–107 mmol/L
Glucose	88 µg/dL	60–140 µg/dL

**Figure 1:**
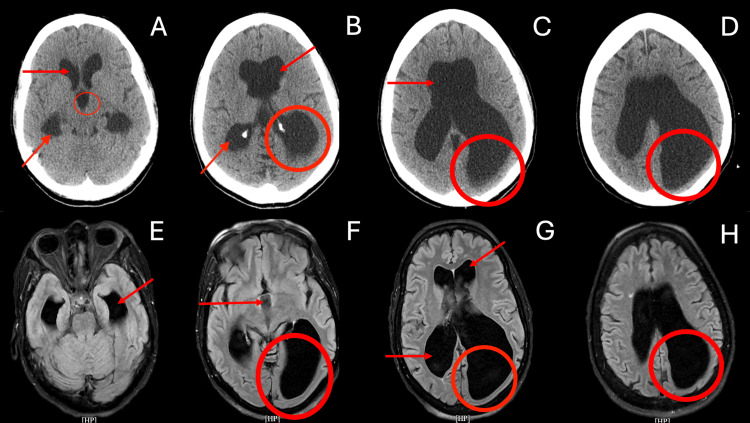
Top row: Head CT scan without contrast. Bottom row: Head MRI T2 with FLAIR. CT overall impression: No acute territorial infarction or acute hemorrhage. An enlarged ventricular system particularly of the lateral ventricles and third ventricle without evidence of dilatation of the fourth ventricle and colpocephaly. A: Arrows show the enlargement of the lateral ventricles at both anterior and posterior locations; the circle shows the enlargement of the third ventricle. B: Arrows demonstrate further enlargement of the lateral ventricles; the circle shows evidence of possible colpocephalic changes. C: The arrow demonstrates severe dilation of the anterior lateral ventricles; the circle shows a clearer *teardrop shape *of the posterior lateral ventricle, showing possible colpocephaly. D: The circle shows continued dilation of the posterior lateral ventricle with colpocephaly. MRI overall impression: No acute infarction, intracranial hemorrhage, or mass lesion. Unchanged dilatation of the ventricular system compared to the previous evaluation. E: The arrow points at enlarged lateral ventricles. F: The arrow shows dilation of the third ventricle; the circle shows severe posterior lateral ventricle dilation. G: Arrows show marked dilation of the lateral ventricles anteriorly and posteriorly; the circle shows evidence of possible colpocephalic changes. H: The circle shows a *teardrop shape*,suggesting possible colpocephaly. CT = computed tomography; MRI = magnetic resonance imaging; FLAIR = fluid-attenuated inversion recovery

At this time, neurosurgery and neurology services were consulted for further workup. Per neurosurgery, no acute intervention was needed, and neurology recommended a magnetic resonance imaging (MRI) of the head to visualize tissue with more detail. CT and MRI of the head are presented in Figure 1. A likely diagnosis of LOVA was considered at this time due to the patient’s previous medical history, complaints, and radiological findings suggesting an arrested congenital process. At this time, the patient was stable, and no repeating seizure episodes occurred. The patient was properly educated on medication adherence, as well as the need for neurological follow-up to monitor her prescription serum levels and seizure activity. The patient understood and agreed to follow-up outpatient at this time.

## Discussion

Hydrocephalus presents a multifaceted challenge in diagnosis and management, particularly in adults. CHiA, encompassing non-acute forms of the condition, includes iNPH as a significant subtype [3,4]. Distinguishing between idiopathic and secondary forms is crucial, given the predominance of iNPH in older adults, while secondary forms may stem from diverse underlying causes. Despite its prevalence, definitive diagnostic findings for NPH often remain elusive, leading to diagnostic hurdles and reliance on CSF shunting responses. However, LOVA differs in both etiology and symptoms from NPH and CHiA.

The heterogeneity of adult ventriculomegaly underscores the varied clinical presentations, ranging from asymptomatic cases to those with significant symptoms [7,8]. Additionally, LOVA’s unique trajectory, originating in childhood and manifesting later in life, contributes to its silent progression until clinical manifestations emerge [11-13].

This variability highlights the necessity for individualized diagnostic and management strategies tailored to each patient’s specific needs. This is especially pertinent in cases where patients remain asymptomatic or stable despite undiagnosed ventriculomegaly. In the case presented, a 52-year-old female with a complex medical history experienced a generalized tonic-clonic seizure, prompting further evaluation to uncover a potential underlying cause.

The absence of focal neurological deficits, urinary incontinence, visual changes, or gait abnormalities complicated the assessment of this case. Nevertheless, despite unremarkable physical examination findings and appropriate serum levels of her anticonvulsant medications, radiological imaging revealed severe ventriculomegaly with signs of colpocephaly. Coupled with reported memory changes and the patient’s age at the time of the breakthrough seizure, this raised suspicion of an atypical presentation of LOVA as the primary diagnosis. This isolated case is a valuable addition to the existing body of knowledge on LOVA, contributing to our understanding of its diverse clinical manifestations.

This case emphasizes the need for a comprehensive approach to evaluating patients with hydrocephalus, considering both clinical symptoms and radiological findings. Despite the absence of typical features associated with hydrocephalus, such as focal neurological deficits or gait abnormalities, the presence of severe ventriculomegaly on imaging, coupled with reported memory changes and the patient’s age at the time of symptom onset, raises suspicion of an atypical presentation of LOVA.

Further research and clinical studies are warranted to enhance our understanding of LOVA and refine diagnostic criteria and management strategies. By expanding our knowledge base and considering atypical presentations such as the one described here, we can improve patient outcomes and provide more targeted and effective care for individuals with hydrocephalus.

## Conclusions

The presented case highlights the complexities involved in diagnosing and managing hydrocephalus, particularly in adult patients. While iNPH is a well-known subtype within CHiA, LOVA presents a distinct clinical entity with unique features and challenges.

The variability in clinical presentations and the silent progression of LOVA from childhood to adulthood underscore the importance of individualized diagnostic and management strategies tailored to each patient’s specific needs. Hydrocephalus has a plethora of etiologies, all of which need to be managed accordingly. It is imperative to determine probable causes such as genetic mutations, aging, alcohol consumption, sleep apnea, hypertension, electrolyte disturbances, and congenital processes, among many others to determine the best possible treatment options.
